# Tarlov cyst with self-healing cauda equina syndrome following combined spinal-epidural anesthesia: a case report

**DOI:** 10.1186/s12871-023-02311-w

**Published:** 2023-10-31

**Authors:** Zhexuan Chen, Chuxi Lin

**Affiliations:** Jieyang People’s Hospital, Jieyang, Guangdong Province China

**Keywords:** Cauda Equina syndrome, Combined spinal-epidural anesthesia, Tarlov cyst

## Abstract

**Background:**

Cauda Equina Syndrome (CES) after Combined Spinal-Epidural Anesthesia (CSEA) is a rare disease that most of the time need surgery to relieve spinal cord compression.

**Case presentation:**

A 34-year-old male patient underwent a procedure for prolapse and hemorrhoids (PPH) under CSEA. Anesthesia and surgery were uneventful. However, the patient gradually experienced urinary retention, lower abdomen and back pain, changes in bowel habits and neurological dysfunction of the lower limbs when the catheter was removed. It was later determined that the patient had Tarlov cyst at the left S1 level in the sacral canal. Finally, the patient completely recovered 20 days after drug conservative therapy onset.

**Conclusion:**

This case suggests that CES might occur even after ordinary CSEA. The risk factors are drug neurotoxicity to ropivacaine and Tarlov cyst, which helped to accumulate ropivacaine. The development of ultrasound-guided CSEA and an ultrasound atlas of the spinal canal are required.

## Background

Cauda equina syndrome (CES) is characterized by at least one or more of the following symptoms: urinary retention, bowel dysfunction, and neurologic deficits in the lower limbs [1]. Few cases of CES have been reported since it is a rare complication that may be caused by lumbar puncture, infection, hematoma, and direct drug neurotoxicity after spinal anesthesia. In addition, it is unusual to recover completely from CES in a short time [2]. Here, we report a case of CES after Combined Spinal-Epidural Anesthesia (CSEA) in a male patient who underwent a procedure for prolapse and hemorrhoids (PPH). The patient experienced urinary retention accompanied by lower abdomen and back pain, changes in bowel habits and neurological dysfunction of the lower limbs. It was later determined that the patient had Tarlov cyst at the left S1 level in the sacral canal. Finally, the patient completely recovered 20 days after drug conservative therapy onset. Written consent was obtained from the patient.

## Case presentation

A 34-year-old man complained of repeated anal mass prolapse with occasional bleeding for five years. The anal mass prolapse after defecation and bleeding was usually bright red and not mixed with the stool. Since the anal mass was exacerbated, he was no longer able to bear the mass and bleeding. The patient received rectosigmoidoscopy and was diagnosed with hemorrhoids. His laboratory work, including routine blood work, electrolyte tests, and hepatic, renal, and coagulation functions, was within the normal range.

After completing the preoperative preparation, the patient was sent to the operating room. The L3/4 intervertebral space was chosen as the puncture point. Cerebrospinal fluid could be seen when the needle is inserted 4 cm. One milliliter of 1% ropivacaine hydrochloride injection (Naropin) mixed with saline solution (0.9 ml) was slowly injected into the subarachnoid space in approximately 20 s. At the same time, an epidural catheter was inserted 8 cm in the L3/4 intervertebral space. After approximately 10 min, anesthesia took effect, and the blocked level of the sensory system was raised and fixed at the T8 level. The duration of PPH operation was 50 min, and the patient had no discomfort during the anesthesia time and operation time.

A patient-controlled epidural analgesia (PCEA) was connected to the epidural catheter. The 100 ml PCEA solution consisted of 2 ml sufentanil citrate injection (YICHANG HUMANWEL PHARMACETICAL, 100 µg/2 ml/bnp), 238.4 mg ropivacaine mesylate injection (PUDE PHARMA, 119.2 mg/bnp) and 98 ml saline solution. The PCEA could last 2 days with a constant injection speed of 2 ml/h. The patient also needed an indwelling urinary catheter for 2 days since he was not able to urinate himself when the PCEA was at work.

However, 2 days later, when we tried to remove the catheter, he was still not able to urinate. At first, we thought it was because of pain, since urinary retention is a common complication of CSEA or PPH. We then administered one celecoxib capsule and one tamsulosin hydrochloride sustained-release capsule per day. After 5 days, the patient tried to remove the catheter the second time, but he still could not urinate and had difficulty defecating with occasional urinary incontinence, and he developed lower abdomen and back pain. This time, levofloxacin hydrochloride tablets and San Jin Pian tablets, were added to his medication list with one and three pills, respectively, administered twice a day. Lactulose oral solution was used to help soften stools. However, his symptoms did not significantly improve.

**Fig. 1:**
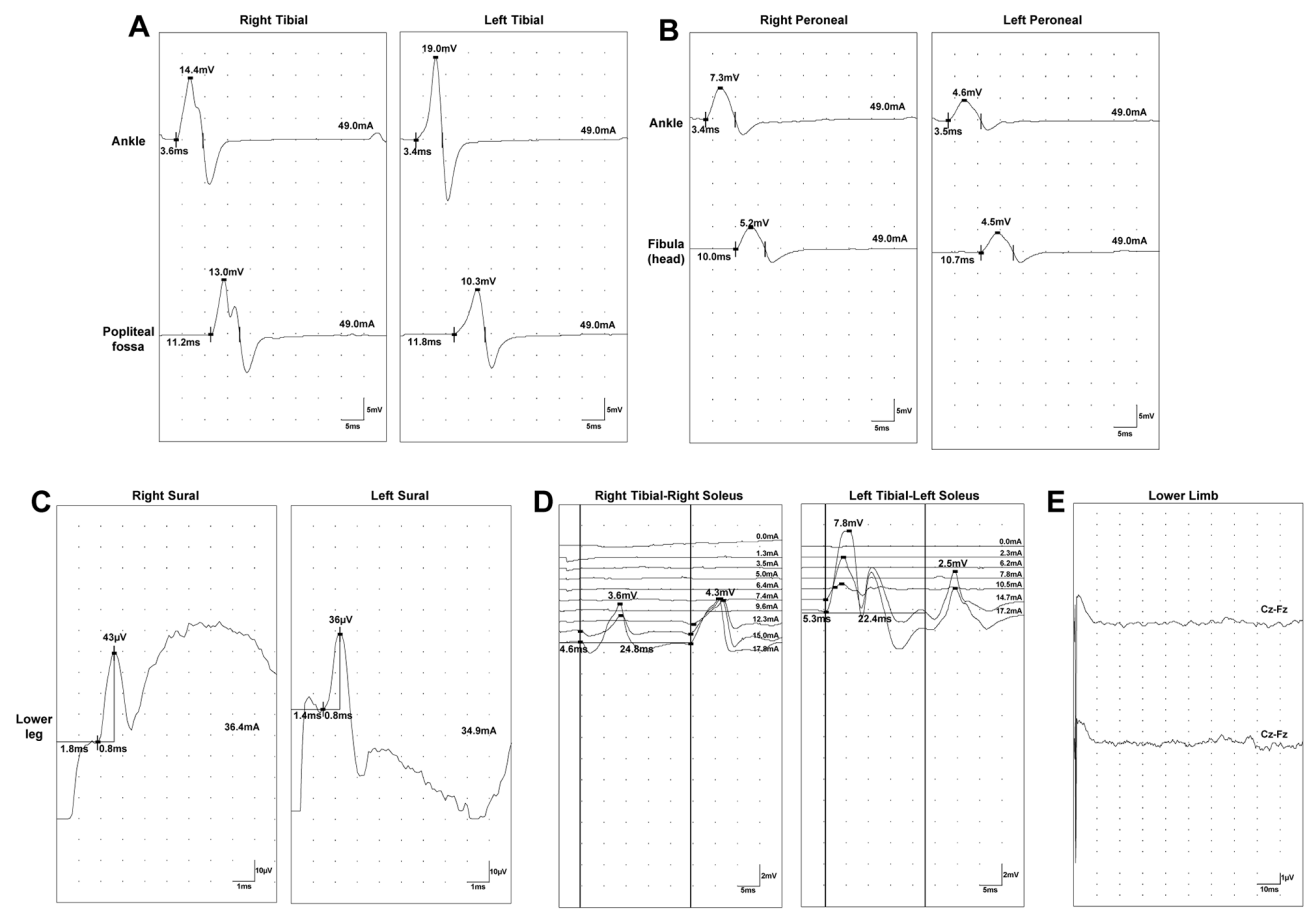
Electromyography of lower limbs Motor nerve conductions of **(A)** tibial nerves and **(B)** peroneal nerves were in normal latency times and with normal amplitudes. Sensory nerve conductions of **(C)** sural nerves were in normal onset latency times and peak latency times and with normal amplitudes. H-reflexes of **(D)** tibial nerves were normal. **(E)** Lower extremity somatosensory evoked potentials P37 and N45 could not be elicited

**Fig. 2:**
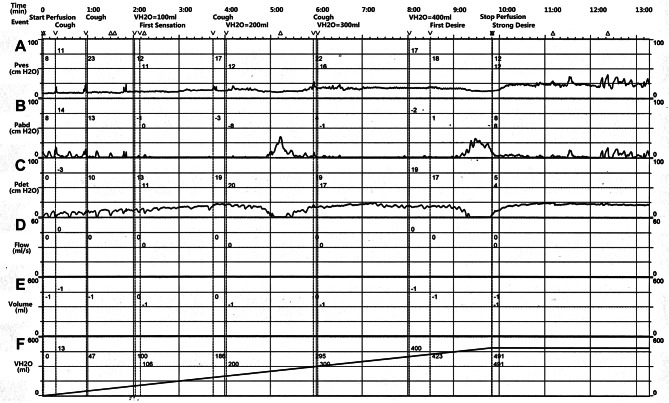
Urodynamic testing With the increase of bladder perfusion, intravesical pressure was maintained at an inadequate level lower than 31 cmH_2_O, even when abdominal pressure was applied. Bladder perfusion was 106 ml at First Sensation and 423 ml at First Desire and 491 ml at Strong Desire. Uroflowmetry and viod volume were not able to be measured. **(A)** Pves: Intravesical pressure (normal range: 31–42 cmH_2_O). **(B)** Pabd: Abdominal pressure. **(C)** Pdet: Detrusor pressure. Pdet = Pves - Pabd **(D)** Flow: Uroflowmetry. **(E)** Volume: Viod volume. **(F)** VH2O: Bladder perfusion

**Fig. 3:**
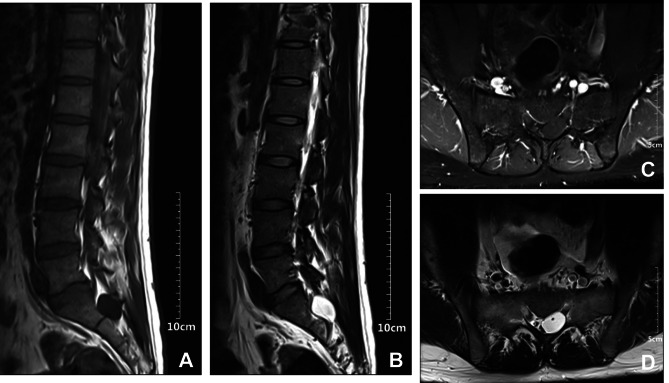
Lumbar magnetic resonance imaging revealed a Tarlov cyst at the left of S1 level in the sacral canal. **(A)** MRI T1 sugittal. **(B)** MRI T2 sugittal. **(C)** MRI T1 axial. **(D)** MRI T2 axial

Further tests were performed on the patient. Electromyography (Fig. 1) showed no abnormal motor or sensory nerve conduction in the lower limbs, but lower extremity somatosensory evoked potentials P37 and N45 were not elicited. In addition, urodynamic testing (Fig. 2) found that the detrusor muscle of his bladder had no contractile force. Uroflowmetry could not be measured, and when abdominal pressure assisted urination, the bladder did not contract. Lumbar magnetic resonance imaging (Fig. 3) showed that there was a 1.79 cm*1.66 cm*1.91 cm Tarlov cyst at the left S1 level in the sacral canal. Although lumbar MRI data showed that the cyst did not compress the lower spinal cord or cauda equine, we still considered that this patient had mild CES.

Based the results, we changed his medications and one methylcobalamin tablet and one flupentixol/melitracen tablet was administered three times and once per day, respectively, to promote nerve growth and relieve tension. Finally, the fourth time we removed the catheter, he was able to urinate by himself after 5 days.

We reviewed him a month later and his urinary retention had completely gone, and his bowel habits had returned to normal. We also performed a sacral ultrasound on the patient, since the cyst was just at the S1 level and might be detected through the intervertebral space at L5/S1 or posterior sacral foramina at S1. However, unfortunately, we could not find the cyst under ultrasound.

## Discussion and conclusions

CES after spinal anesthesia is very rare, with an incidence of 0.0027% [2, 3], and is associated with varying degrees of symptoms, including bowel and bladder dysfunction, lower abdomen and back pain, insensate perineal areas and neurologic deficits in the lower limb [4]. Motor dysfunction, sensory dysfunction and abnormal evoked potentials are classified as neurologic deficits in the lower limb [5]. Bladder dysfunction is characterized by urinary retention, which is the most common and easily detectable symptom of CES [1]. The literature reported that more than two-thirds of CES would result in permanent neurological deficits and seldom cases could completely recover within a month [6]. The etiologies of CES after spinal anesthesia may occur due to direct or indirect trauma, infection, drug neurotoxicity and spinal cord compression by hematoma, lumbar artery pseudoaneurysm or perineural cysts [7].

In the present case, a healthy young man underwent a successful operation, and urinary retention occurred just after the end of PCEA. Since urinary retention was thought to be a common and self-limiting complication of CSEA or PPH [8], we did not pay too much attention to it at first. However, further symptoms, such as bowel dysfunction and lower abdomen and back pain, gradually emerged. Although he did not experience motor or sensory dysfunction in the lower limbs or perianal area, electromyography showed lower extremity somatosensory evoked potentials P37 and N45 were not elicited. Interestingly, a Tarlov cyst was found at the left S1 level in the sacral canal through MRI. All these symptoms indicated that he should have CES, and the Tarlov cyst must have an effect on his symptoms [4].

Ishiguro et al. described CES associated with S2 root cysts in a patient who underwent a prosthetic femoral removal surgery [9]. Finally, the patient underwent S1 to S5 laminectomy to reduce spinal flow blockage due to stenosis and the laminectomy completely eliminated sensation loss and urinary incontinence. However, in the current case, there was no spinal stenosis or cauda equina nerve root compression by the S1 nerve root sheath cyst.

Another important risk factor for CES after uneventful CSEA is drug neurotoxicity of local anesthetics [10]. Ropivacaine was reported to result in CES after epidural anesthesia [11]. Epidural injection could result in cystic accumulation of injected local anesthetics [2]. In this way, local anesthetics, including ropivacaine, might cause a mass effect from fluid accumulation, which finally leads to CES [12]. Therefore, based on the patient’s clinical course and MRI findings, the etiologies of CES were drug neurotoxicity to ropivacaine combined with Tarlov cyst, which helped to accumulate ropivacaine.

CES is reported to be the second most frequent neurological complication of spinal anesthesia [6]. But if we could obtain early diagnosis of the Tarlov cyst before CSEA, we could make changes on an upper puncture point, less anesthetic, no PCEA, or intravenous anesthesia. Ultrasound guidance can visualize the injection needle and provide real-time vision during anesthesia [13]. However, when we performed sacral ultrasound on the patient to find Tarlov cyst at the S1 level using Doppler ultrasound, we could not discover anything through the L5/S1 intervertebral space or S1 posterior sacral foramina. Recent studies show that lumbar ultrasound has been relatively well established and applied to spinal anesthesia [14], but only one research related to sacral ultrasound was published [15]. As research progresses, we believe that an ultrasound atlas of the spinal canal, which is essential to the growth of an anesthesiologist and necessary for the development of precision anesthesia, will be available in the near future.

In summary, we report a case of CES after uneventful CSEA. The risk factors for the current case are drug neurotoxicity to ropivacaine and Tarlov cyst, which helped to accumulate ropivacaine. Although our patient did not undergo a second operation and completely recovered within 20 days, early detection and treatment of CES were also essential to minimize the risk of permanent damage. We require that the development of ultrasound-guided CSEA is necessary to avoid CES, not only to help anesthesiologists diagnose relative contraindications before CSEA but also to guide the needle to avoid injury during CSEA.

## Data Availability

The datasets used and/or analysed during the current study available from the corresponding author on reasonable request.

## Abbreviations

CES: Cauda Equina Syndrome
CSEA: Combined Spinal-Epidural Anesthesia
PPH: procedure for prolapse and hemorrhoids
PCEA: patient-controlled epidural analgesia
